# Increased serum concentration of apolipoprotein B is associated with an increased risk of reaching renal replacement therapy in patients with diabetic kidney disease

**DOI:** 10.1080/0886022X.2020.1745235

**Published:** 2020-04-03

**Authors:** Wen-bo Zhao, Lin Zhu, Tohty Rahman

**Affiliations:** a Department of Nephrology, The Third Affiliated Hospital of Sun Yat-Sen University, Guangzhou, China; b Department of Nephrology, The First Affiliated Hospital of Jinan University, Guangzhou, China; c Zhongshan School of Medicine, Sun Yat-Sen University, Guangzhou, China

**Keywords:** Diabetic kidney disease, chronic kidney disease, apolipoprotein B, renal replacement therapy

## Abstract

**Objective:**

Few studies have investigated the association of apolipoprotein B (ApoB) with the progression of diabetic kidney disease (DKD) and the risk of renal replacement therapy (RRT).

**Method:**

In this retrospective cohort study, a group of 258 DKD patients with stage 3–5chronic kidney disease(CKD)were divided into low ApoB (<1.1 g/L) and high ApoB (≥1.1 g/L) groups and followed-up for 20.51 ± 6.11 months. The association of the serum ApoB concentration with RRT was determined by Kaplan–Meier and Cox regression analysis. ApoB was measured in the serum.

**Results:**

Ninety-three of the 258 DKD patients needed RRT during follow-up. Kaplan–Meier analysis showed that patients with high ApoB were significantly more likely to progress to RRT than those with low ApoB (log-rank = 16.62, *p* < 0.001). The presence of high ApoB increased the risk of RRT. Analysis of ApoB as either a categorical (<1.1 g/L or ≥1.1 g/l) or continuous variable by univariate and multivariate regression found that ApoB was an independent risk factor of DKD progression to RRT in this group of DKD patients with stage 3–5 CKD (*p* < 0.05).

**Conclusion:**

Increased ApoB was an independent predictor of progression to RRT. A larger study is needed to confirm the unfavorable prognosis of increased ApoB in DKD patients.

## Background

Chronic kidney disease (CKD) leads to loss of renal function. It is a public health problem worldwide, and the estimated prevalence in adults in the Chinese population is 10.8% [1,2]. The identification of factors that increase the risk of CKD and management of early-stage disease may slow or prevent the progression to end-stage renal disease. Dyslipidemia is a common diagnosis in CKD patients and it affects renal function both directly and indirectly by provoking systemic inflammation, vascular injury, oxidative stress, and activation of various molecular signaling pathways [3–5]. Decreased renal function is associated with the disruption of lipoprotein metabolism, dyslipidemia, and the accumulation of atherogenic particles [6,7]. Serum lipid abnormalities may be independent risk factors for CKD progression. Apolipoprotein (Apo) B present in the serum is composed of two subunits, ApoB 100 and ApoB 48. It transports plasma lipids and is required for the formation of various plasma lipoprotein particles. ApoB is increased at all stages of CKD, and as catabolism of very low-density lipid (VLDL)-ApoB-100 is slowed in CKD, accumulation of ApoB-containing lipoproteins may result primarily from decreased clearance rather than from increased synthesis [8–10]. In animal studies, hyperlipidemia was found to increase glomerulosclerosis and tubulointerstitial damage leading to the development and progression of kidney damage [11,12]. Some, but not all, studies have found that ApoB and low-density lipid cholesterol (LDL-C) were independently associated with CKD progression [13–16]. The Chronic Renal Insufficiency Cohort Study (CRIC) study found that blood lipids were not independently associated with CKD progression. The ApoB/A1 ratio was associated with CKD progression, but ApoB alone was not [17]. A retrospective study of over 10,000 healthy participants did not find a longitudinal association between incident CKD and baseline ApoB or ApoB/ApoA1, and in a study in hospitalized heart failure patients, ApoA1 and ApoB were not associated with renal dysfunction [18,19]. Few studies have investigated the association between ApoB and the decline in kidney function in diabetic kidney disease (DKD) patients. Dalrymple et al. [20] reported that the use of lipid-lowering drugs postponed decrease of the estimated glomerular filtration rate (eGFR) in diabetes patients with increased serum ApoB and proteinuria. However, the association between increasing ApoB and the risk of renal replacement therapy (RRT) was not studied. This study analyzed the association between ApoB and RRT in DKD patients with stage 3–5 CKD by Kaplan–Meier analysis and Cox regression.

## Subjects and methods

### Study design and patients

This retrospective cohort study evaluated the medical records of 258 adult DKD patients with stage 3–5 CKD who were patients at the Third Affiliated Hospital of Sun Yat-Sen University between December 2010 and December 2014. The patients were followed in the outpatient renal clinic of the Sun Yat-Sen University Hospital. The study protocol was approved by the institutional review board of the Third Affiliated Hospital of Sun Yat-Sen University (code: [2013]2–75). Written informed consent for each participant was waived.

Patients were diagnosed following the 1999World Health Organization diabetes criteria and the estimated glomerular filtration rate (eGFR) and urinary microalbumin DKD criteria included in the NKF-KDOQI Guidelines [21,22]. The Chronic Kidney Disease Epidemiology Collaboration (CKD-EPI) equation was used to calculate the estimated glomerular filtration rate (eGFR) in mL/min/1.73 m^2^. ApoB was assayed in the serum [23]. RRT was defined as initiation of peritoneal dialysis or hemodialysis, and the reasons for starting RRT as follows: Fluid overload was difficult to correct with diuretics, hypertension was difficult to correct with antihypertensive drugs, and metabolic acidosis persisted despite medical treatment. Some patients experienced uremic encephalopathy, pericarditis or pleurisy, severe bleeding, continuous vomiting, or malnutrition.

The mean patient age was 66.13 ± 11.88 (range, 27–91) years of age, 156 were men and 102 were women. The cohort was divided into 165 non-RRT and 93 RRT patients. The study endpoint was the time from baseline to RRT. The average follow-up was 20.51 ± 6.11 months. The study cohort was also divided into low ApoB (<1.1 g/L) and high ApoB (≥1.1 g/L) groups, with 1.1 g/L as the upper limit of normal. Patients < 18 years of age, with type 1 diabetes, a history of donating or receiving a kidney, primary kidney disease, full or partial loss of a kidney, diabetic ketosis, hyperosmolar coma, fever, heart failure, infection, secondary hypertension, or severe hypohepatia were excluded from the analysis. The pathogenesis and factors affecting the progression of type 1 diabetes and primary kidney disease are different from those of type 2diabetes, so they are not included in our study. Patient variables retrieved from their electronic medical records included sex, age, race, and medical history. Laboratory values included hemoglobin, phosphate, cholesterol, triglycerides potassium, albumin, urinary albumin-to-creatinine ratio (UACR) and eGFR, and other the specific lab values. Physiological data included DKD-associated complications and comorbid diseases including diabetic retinopathy, hypertension history, and cardiovascular disease.

### Statistical analysis

Statistical analysis was performed with SPSS 20.0 (IBM Corp., Armonk, NY, USA). Normally distributed data were reported as means ± standard deviation. Continuous variables with a normal distribution were reported as medians and interquartile range, and the significance of differences was determined by the Kolmogorov–Smirnov test. Categorical variables were reported as numbers and percentages. Continuous variables that were not normally distributed were analyzed with the Kruskal–Wallis rank-sum test. Categorical variables, including differences between the low ApoB and high ApoB groups, were analyzed with the chi-squared test. Cumulative risk of need for RRT was estimated by the Kaplan–Meier method. The significance of the association between ApoB and RRT was analyzed in univariate and multivariate Cox regression models with patient variables as covariates in high and low ApoB patients separately and also in models considering ApoB as a continuous variable. Differences with *p*-values <0.05 were considered statistically significant.

## Results

### Patient characteristics

The characteristics and clinical data of the 258 patients are summarized in Table 1. The low ApoB group included 150 cases and the high ApoB group included 108. The participants in the low ApoB group were significantly older than those in the high ApoB group, and their HDL-C was significantly higher. Serum phosphate concentration, HbA1c, TG, TC, and LDL were significantly higher in the high ApoB than in the low ApoB group. Both ApoB and ApoA1 were increased in the high compared with the low ApoB participants. Between-group differences in smoking history, body mass index (BMI), hypertension, diabetic retinopathy, lipid-lowering drugs, UACR and eGFR were not significant.

**Table 1.** Characteristics of patients with ApoB levels below and above the 1.1 g/L upper limit of normal.

Variables	Low ApoB	High ApoB	z/χ^2^	*p* Value
	group (*n* = 150)	group (*n* = 108)		
Gender				
Male (%)	99 (66.00)	57 (52.78)	4.592	0.039
Female (%)	51 (34.00)	51 (47.22)		
Age (years), median (IQR)	70 (61, 77)	64.00 (57.25, 70.75)	−3.684	<0.001
Smoking history (%)	32 (21.33)	29 (26.85)	1.059	0.373
BMI/(kg/m^2^) median (IQR)	23.76 (21.96, 26.98)	24.79 (22.91, 26.83)	−1.52	0.129
Hypertension (%)	127 (84.67)	98 (90.74)	2.077	0.187
cardiovascular disease (%)	35 (23.33)	24 (22.22)	0.044	0.881
Diabetic retinopathy (%)	30 (20.00)	21 (19.44)	0.012	1
HbA1C(%), median (IQR)	7.10 (6.40, 8.05)	7.75 (6.60, 9.10)	−2.03	0.042
HGB/(g/L), median (IQR)	108.00 (94.00, 123.00)	114.00 (97.25, 127.75)	−1.751	0.08
TC/(mmol/L), median (IQR)	4.28 (3.41, 4.97)	5.30 (4.49, 6.36)	−7.153	<0.001
TG/(mmol/L), median (IQR)	1.32 (1.02, 1.91)	2.41 (1.73, 3.74)	−7.898	<0.001
LDL-C/(mmol/L), median (IQR)	2.37 (1.78, 3.01)	3.05 (2.33, 3.91)	−4.693	<0.001
HDL-C/(mmol/L), median (IQR)	0.99 (0.85, 1.35)	0.95 (0.81, 1.15)	−2.003	0.045
ApoA/(mmol/L), median (IQR)	1.19 (1.01, 1.43)	1.29 (1.16, 1.46)	−2.492	0.013
ApoB/(mmol/L), median (IQR)	0.81 (0.70, 0.94)	1.42 (1.23, 1.67)	−13.699	<0.001
LPa/(g/L), median (IQR)	173.80 (91.55, 348.50)	201.25 (82.50, 425.13)	−1.027	0.305
Albumin/ (g/L), median (IQR)	37.15 (33.93, 40.10)	38.15 (34.93, 40.98)	−1.017	0.309
Ca/(mmol/L), median (IQR)	2.27 (2.16, 2.39)	2.31 (2.19, 2.40)	−1.834	0.067
P/(mmol/L), median (IQR)	1.17 (1.06, 1.31)	1.25 (1.14, 1.39)	−3.439	0.001
Fasting blood glucose /(mmol/L), median (IQR)	5.96 (4.64, 7.43)	5.94 (4.78, 7.92)	−0.687	0.492
Uric acid/(μmol/L), median (IQR)	452.25 (355.73, 554.38)	470.30 (390.00, 573.25)	−1.476	0.14
Creatinine/ (μmol/L), median (IQR)	159.50 (122.00, 252.58)	152.15 (128.25, 152.15)	−0.129	0.898
BUN/(mg/L), median (IQR)	10.31 (7.67, 15.96)	9.12 (7.46, 13.04)	−1.932	0.053
CysC/(mg/L), median (IQR)	1.95 (1.55, 2.86)	1.98 (1.57, 2.60)	−0.136	0.892
Diuretic (%)	34 (22.67)	18 (16.67)	1.405	0.272
Lipid-lowering drugs (%)	75 (50.0)	68 (62.96)	4.271	0.403
UACR(mg/g), median (IQR)	1184.10 (251.59, 2481.64)	1234.55 (373.21, 2402.66)	−0.457	0.647
eGFR/[mL/(min·1.73m^2^)], median (IQR)	40.24 (25.62, 51.38)	42.04 (28.22, 50.46)	−0.165	0.869

eGFR: estimated glomerular filtration rate; BMI: body mass index; HGB: hemoglobin; Scr: serum creatinine; BUN: nlood urea nitrogen; UA: uric acid; CysC: cystatin C; TC: total cholesterol; TG: triglycerides; HDL: high-density lipoprotein; LDL: low-densitylipoprotein; ApoA: apolipoprotein A; ApoB: apolipoprotein B; Lpa: lipoprotein a; Ca: serum calcium; P: serum phosphorus; ALB: serum albumin; HbA1c: glycosylated hemoglobin; FPG: fasting plasma glucose; UACR: urinary albumin to creatinine ratio.

### Kaplan–Meier and multivariate-adjusted cox regression analysis

**Figure 1.**
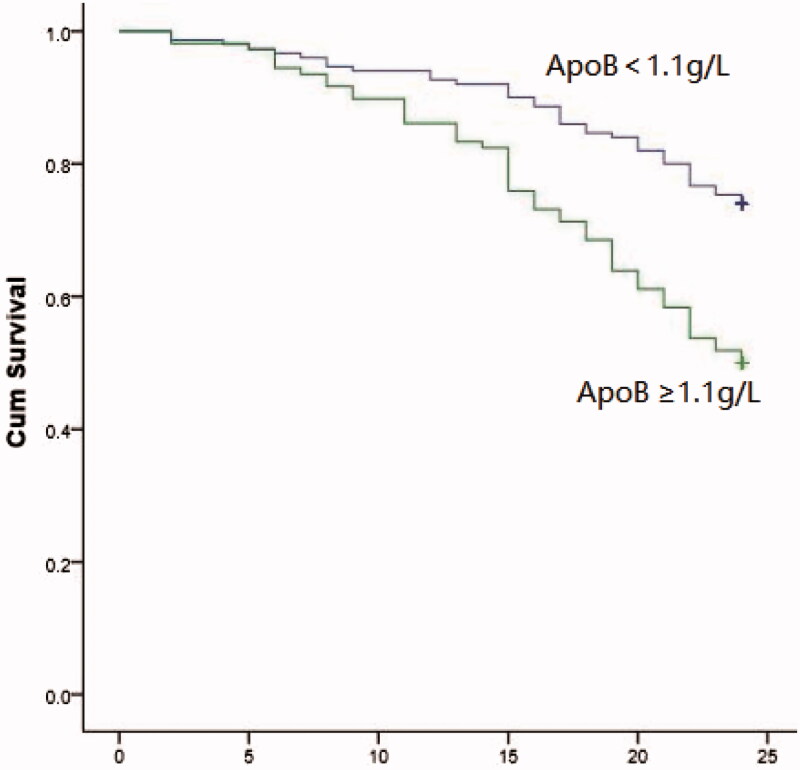
Kaplan–Meier renal survival curves for high-ApoB group VS. low-ApoB group.

Ninety-three of the 258 patients (36%) required RRT during a mean follow-up of 20.51 ± 6.11 months, 54 (58%) in the high ApoB group and 39 (42%) in the low ApoB group (*p* < 0.001). Kaplan–Meier analysis found that patients with high ApoB were significantly more likely to progress to RRT than those with low ApoB (log-rank = 16.62, *p* < 0.001; Figure 1), Cox regression analysis of the association between high ApoB versus low ApoB and progression of DKD to RRT is shown in Table 2. Unadjusted univariate analysis yielded a hazard ratio (HR) of 2.282 (*p* < 0.001). Model 1was corrected for gender, age, BMI, smoking history, history of hypertension, cardiovascular disease, retinopathy (HR = 2.252, *p*<0.001). Model 2 was corrected for, HGB, HbA1c, ALB, Ca, P, FBS, BUN, CYC, UA, TC, TG, HDL, LDL, ApoA, lysophosphatidic acid (LPA), UACR, and eGFR in addition to Model 1 (HR = 2.796, *p*<0.001). Model 3 was corrected for Model 2 variables plus diuretics and lipid-lowering drugs (HR =3.241, *p* < 0.001, Table 2). The association between ApoB considered as a continuous variable and the risk of DKD progressing to RRT was analyzed by multi-model Cox regression (Table 3). The adjusted HR for the univariate model was 2.157 (95% CI:1.438–3.237, *p* < 0.001). The adjusted HRs were 2.157 (95% CI:1.438–3.237, *p* < 0.001) for Model 1, 3.161 (95% CI: 1.990–5.019, *p* < 0.001) for Model 2, and 3.161(95% CI: 1.990–5.019, *p* < 0.001) for Model 3.

**Table 2.** Univariate and multivariate Cox regression models of RRT risk in patients with high ApoB and low ApoB levels.

	Univariate model	Model 1	Model 2	Model 3
HR	2.282 (1.510, 3.447)	2.252 (1.458, 3.479)	2.796 (1.737, 4.499)	3.241 (2.038, 5.155)
*p* Values	<0.001	<0.001	<0.001	<0.001

HR: hazard ratios; Univariate model: unadjusting relevant factors; Model 1: univariate model plus gender, body mass index, smoking history, hypertension history, cardiovascular disease, retinopathy lesion; Model 2: Model 1 plus hemoglobin, glycosylated hemoglobin, serum albumin, serum calcium, serum phosphorus, fasting plasma glucose, cystatin C, uric acid, total cholesterol, triglycerides, high-density lipoprotein, low-densitylipoprotein, apolipoprotein A, lipoprotein a, urinary albumin to creatinine ratio, estimated glomerular filtration rate; Model 3: Model 2 plus diuretics and lipid-lowering drugs.

**Table 3.** Univariate and multivariate Cox regression models of RRT risk for ApoB as a continuous variable.

Model	B	SE	Wald	df	Significance	HR	95.0% CI for Exp (B)	
							Lower	Upper
Univariate model	0.769	0.207	13.785	1	0.000	2.157	1.438	3.237
Model 1	0.769	0.207	13.785	1	0.000	2.157	1.438	3.237
Model 2	1.151	0.236	23.782	1	0.000	3.161	1.990	5.019
Model 3	1.151	0.236	23.782	1	0.000	3.161	1.990	5.019

HR: hazard ratios; Univariate model: unadjusting relevant factors; Model 1: univariate model plus gender, age, body mass index, smoking history, hypertension history, cardiovascular disease, retinopathy lesion; Model 2: Model 1 plus hemoglobin, glycosylated hemoglobin, serum albumin, serum calcium, serum phosphorus, fasting plasma glucose, cystatin C, Uric acid, total cholesterol, triglycerides, high-density lipoprotein, low-densitylipoprotein, apolipoprotein A, lipoprotein a, urinary albumin to creatinine ratio, estimated glomerular filtration rate; Model 3: Model 2 plus diuretics and lipid-lowering drugs.

## Discussion

To the best of our knowledge, the relationship between ApoB and progression of DKD to RRT has not been previously described. Kaplan–Meier analysis and multifactor Cox regression found that ApoB, as both a categorical (<1.1 g/L versus ≥1.1 g/L) and a continuous variable, was a risk factor of DKD progression. An increase of serum ApoB was an independent predictor of DKD progression to RRT in this study group within 2 years of follow-up, and patients with high baseline ApoB had a higher risk of RRT than those with low baseline ApoB.

DKD is a chronic microangiopathy of diabetes. The primary causes are renal arteriosclerosis, glomerular sclerosis caused by arteriolonephrosclerosis, and renal microvascular lesions. The impairment of renal function results from the effects of dyslipidemia on vascular mesangial and renal tubular cells [24]. Lipid-lowering therapy can slow the progression of kidney disease and the rate of decline in renal function in diabetes patients. A study by Colhoun et al. [25] found that statin therapy protected renal function in patients with diabetes and proteinuria and slowed the decline of the eGFR. ApoB is composed of two subunits, ApoB 100 and ApoB 48. It transports plasma lipids and is required for the formation of various plasma lipoprotein particles. Increased Apo B is predictive of atherosclerotic cardiovascular disease [26,27]. ApoB increases in CKD patients are correlated with microalbuminuria and progression to overt nephropathy in type 2 diabetes, and renal deposition of ApoB accelerates the progression of glomerulosclerosis [8,28,29]. A recent review by Tabas et al. [30] reported that a long-term disturbance of carbohydrate metabolism can increase blood ApoB and lipids with subendothelial accumulation of ApoB-containing lipoproteins, activation of cytokines, such as interleukin, platelet-derived growth factor, insulin-like growth factor-1(IFG-1), inflammatory responses, and necrosis.

In conclusion, increasing ApoB has until now not been correlated with the progression of CKD. In this study, increased and increasing ApoB were found to be independently associated with progression of CKD in diabetes patients until RRT was needed. The increase of ApoB had an unfavorable prognosis. The study was limited by its small patient enrollment. Although the relationship between ApoB concentration and progression to RRT remained strong after adjusting for relevant covariates in multivariate analysis, residual confounding and variables that were not evaluated may have contributed to this finding. Large-scale clinical confirmation is needed.
